# How will climate change affect endangered Mediterranean waterbirds?

**DOI:** 10.1371/journal.pone.0192702

**Published:** 2018-02-13

**Authors:** Francisco Ramírez, Carlos Rodríguez, Javier Seoane, Jordi Figuerola, Javier Bustamante

**Affiliations:** 1 Department of Wetland Ecology, Estación Biológica de Doñana (CSIC), C/ Américo Vespucio 26, Sevilla, Spain; 2 Departament de Biologia Evolutiva, Ecologia i Ciències Ambientals, Universitat de Barcelona, Facultat de Biologia, Avda. Diagonal 643, Barcelona, Spain; 3 Department of Conservation Biology, Estación Biológica de Doñana (CSIC), C/ Américo Vespucio 26, Sevilla, Spain; 4 Terrestrial Ecology Group (TEG). Departamento de Ecología. Universidad Autónoma de Madrid, Madrid, Spain; 5 Remote Sensing and GIS Lab (LAST-EBD). Estación Biológica de Doñana (CSIC), C/ Américo Vespucio 26, Sevilla, Spain; Sichuan University, CHINA

## Abstract

Global warming and direct anthropogenic impacts, such as water extraction, largely affect water budgets in Mediterranean wetlands, thereby increasing wetland salinities and isolation, and decreasing water depths and hydroperiods (duration of the inundation period). These wetland features are key elements structuring waterbird communities. However, the ultimate and net consequences of these dynamic conditions on waterbird assemblages are largely unknown. We combined regular sampling of waterbird presence through one annual cycle with in-situ data on relevant environmental predictors of waterbird distribution to model habitat selection for 69 species in a typical Mediterranean wetland network in southwestern Spain. Species associations with environmental features were subsequently used to predict changes in habitat suitability for each species under three climate change scenarios (encompassing changes in environmental predictors that ranged from 10% to 50% change as predicted by regional climatic models). Waterbirds distributed themselves unevenly throughout environmental gradients and water salinity was the most important gradient structuring the distribution of the community. Environmental suitability for the guilds of diving birds and vegetation gleaners will decline in future climate scenarios, while many small wading birds will benefit from changing conditions. Resident species and those that breed in this wetland network will also be more negatively impacted than those using this area for wintering or stopover. We provide a tool that can be used in a horizon-scanning framework to identify emerging issues in waterbird conservation and to anticipate suitable management actions.

## Introduction

Wetlands have some of the highest biodiversity and biological productivity levels in the world [1,2], and several globally threatened species largely depend on them [1,3,4]. Although many of the world’s most important wetlands are protected, they are also affected by a range of human and climate-driven impacts that may threaten their biodiversity and associated ecosystem services [2,5–8]. For instance, human activities have resulted in extensive wetland fragmentation, modification and loss [4,9–12]. The impact on wetlands has been exacerbated by the natural insularity of these patchy habitats, which are surrounded by a terrestrial matrix [13,14]. Ultimately, this has resulted in biodiversity loss rates that far exceed those of other, more terrestrial ecosystems [1,6,15].

Concurrently, ecological and hydrological impacts resulting from climate change may pose additional, cumulative threats for wetland ecosystems [2,16]. This is because direct anthropogenic impacts, such as water extraction and pollution, may be exacerbated by a climate-driven reduction in the water budget (i.e., increasing evapotranspiration and decreasing precipitation regimes), as predicted for mid-latitude regions such as the Mediterranean basin [17]. Together, both anthropogenic and climatic stressors are expected to affect Mediterranean wetlands [2,8], which, in turn, are highlighted as global biodiversity hotspots that should be prioritized for conservation [18].

Waterbirds have become a ‘flagship community’ for leveraging management strategies for the conservation of wetlands, especially under the current context of rapid environmental change [2,19]) and habitat and biodiversity loss [1,12,20]. In part, this is because waterbird communities, which are composed of species with different ecological needs and conservation requirements, are extremely sensitive to changes in the availability of suitable and heterogeneous wetland habitats [15,21–25]. The net consequences of climate and human impacts for the waterbird community remain unclear, however, as responses to environmental perturbations are expected to be species-specific [4,8,25,26] and even site-specific [6,8,27]. Seasonal migratory movements may complicate this picture, as wetlands are used at different times by different waterbird species for reproduction, migratory stopover, or winter refuge over the course of their annual cycles [28,29]. Environmental conditions can also fluctuate seasonally [30,31], thus resulting in varying resource and habitat availability for waterbirds throughout the year [29]. Finally, species co-occurring in space and time throughout the annual cycle may have different conservation requirements [32]. Thus, prospective exercises aimed at horizon-scanning waterbird responses to climate- and human-driven environmental changes should combine accurate knowledge of environmental factors structuring waterbird communities with knowledge about species requirements throughout the annual cycle [23,33].

Here, we investigated waterbird distribution and association with habitat features in a wetland network in southwestern Spain. For this purpose, we combined regular sampling of waterbird occurrences at fixed localities throughout a complete annual cycle with in-situ data measurements of relevant environmental variables. Owing to the complex and dynamic nature of these wetlands, local environmental variables are expected to be better predictors of waterbird presence than more general, large-scale and often static topo-climatic or average environmental variables [34,35]. In turn, our study area, which includes the Doñana wetland complex, provides an ideal case study for assessing the impacts of anthropogenic and climate stressors on waterbird populations. The wetland network is one of the most important breeding sites for waterbirds in Europe, but also a key stopover and wintering hotspot for migratory waterbirds coming from Central and Northern Europe [26,27,32,36]. Further, human and climate impacts on wetlands are particularly severe in this Mediterranean region [37–40], a trend that will likely continue and further exacerbate pressures on wetland biodiversity well into the future [2]. Accordingly, we framed our results within the current global warming trend that is expected to cause changes in wetland conditions (e.g., increasing salinity and temporality of water bodies) by predicting changes in waterbird occurrence, and hence in habitat suitability, within different scenarios of climate change. In particular, we fitted statistical models to the habitat selection of individual species of waterbirds in this typical Mediterranean wetland network along an annual cycle. We aimed to predict how habitat suitability for each species would improve or deteriorate under three climate change scenarios within the range of predictions of regional climatic models for the next hundred years.

## Material and methods

### Study area and data collection

**Fig 1:**
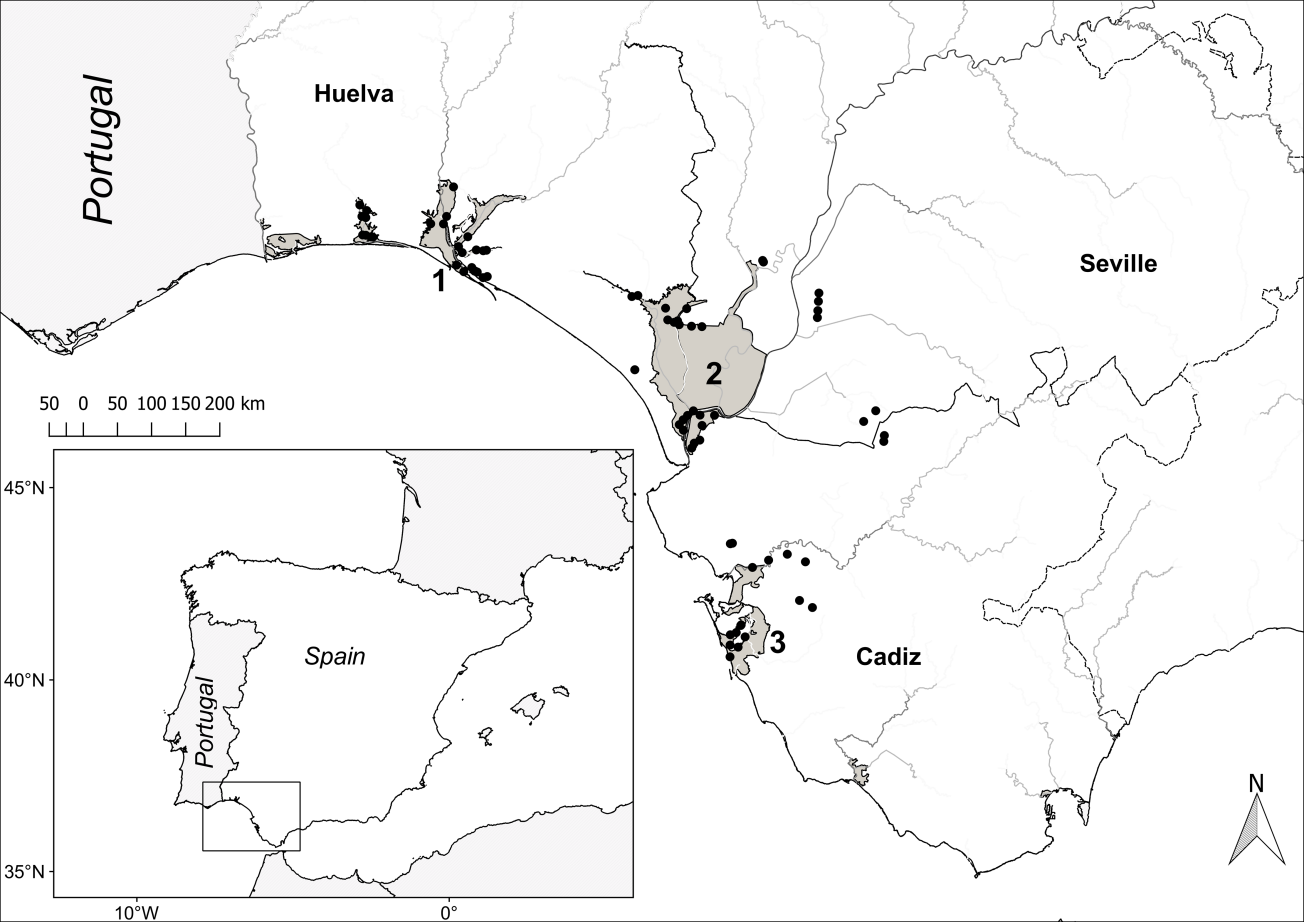
Study area. Point-counts were carried out fortnightly at 80 different localities (black dots) within an area of ca. 6,000 km^2^ in southwestern Spain that encompasses permanent and temporary water masses within the provinces of Huelva, Cadiz and Seville. This wetland network includes the Tinto & Odiel marshes (1), the Doñana wetland complex (2) and Bay of Cadiz (3).

**Fig 2:**
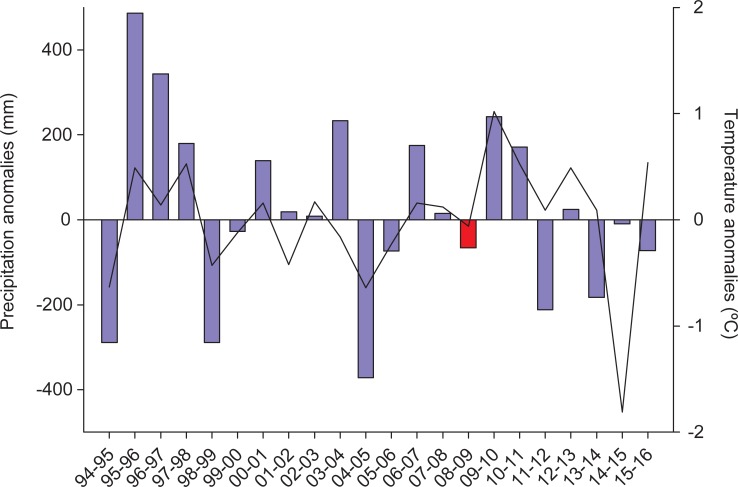
Meteorological conditions in the study area. Yearly anomalies (deviations from the long-term -1994 to 2016- mean) in the annual accumulated precipitation (blue bars, in mm) and the yearly-averaged daily mean temperature (black line, in °C). Red bar indicates our sampling year, i.e. the year in which our point-counts were carried out.

Fieldwork was conducted in a wetland network in southwestern Spain encompassing ca. 6,000 km^2^ (Fig 1). This network included permanent and temporary waterbodies in a salinity gradient ranging from fresh water ponds to brackish marshes to salt pans, in the provinces of Huelva, Seville and Cadiz. The climate is Mediterranean sub-humid with rainy winters and dry summers. The study area includes the Doñana wetland complex, a large and shallow floodplain at the estuary of the Guadalquivir River. This complex is considered the Western Europe’s largest sanctuary for migratory birds and holds a number of national and international recognitions (National Park, biosphere reserve, Ramsar site) [41]. We carried out fortnightly surveys (1718 point-counts) at 80 different fixed selected localities (Fig 1) throughout the 2008–2009 annual cycle (from January 2008 to February 2009). With an annual accumulated precipitation of 476 mm and an average daily mean temperature of 17.2°C, this annual cycle fell well within the average range for the study area (541 mm and 17.3°C for the long term 1994–2016 average; Fig 2). Waterbird species occurrences (presence/absence) were recorded during a 20-minute interval at each site scanning a 180° field-of-view in front of the observer. The direction of observation at the locality was selected to cover the wetland habitat with homogeneous characteristics. Concurrently, the observer recorded environmental information and relevant predictors in the observed area likely driving both the detectability and the presence of waterbirds (Table 1).

**Table 1:** Predictors and control factors. Complete list of predictors and control factors considered for modelling habitat associations in the waterbird community in the southwestern Spain wetland network.

**WATERBIRD DETECTABILITY**	
Observer (two-level factor)	*Two different observers that alternate point-counts among localities and throughout the study period*
Visibility (three-level factor)	*Good*, *medium or poor visibility (as appreciated by the observer)*
Meteorology (four different two-level factors)	*Occurrence (presence/absence) of sun*, *clouds*, *rain*, *and fog*
Day time (covariate -4 d.f. spline-)	*Point-count starting time*, *to account for waterbirds' circadian rhythms*
**WATERBIRD PRESENCE**	
**Control factors**	
Date (covariate -4 d.f. spline-)	*Days from January 1*^*st*^, *to account for seasonal changes in the occurrences of migratory waterbirds*
**Geographic predictor**	
Geographic locations (two covariates)	*Latitude and longitude*
Distance to coastline (covariate)*Minimum Euclidean distance to the coastline*	
**Environmental predictor**	
Water (two-level factor)	*Occurrence (presence/absence) of water in the wetland (to consider for the drying up of seasonal waterbodies)*
Isolation (covariate)	*% of wetlands within a 10 km buffer from the point-count locality*
Hydroperiod (covariate)	*% of surveys with presence of water in the wetland*
Relative flooded area (covariate)	*% of flooded area relative to maximum flooded area at the locality*
Salinity (six covariates)*Water and soil conductivity (as recorded at the locality on each sampling date*, *annual mean*, *sd and coefficients of variations–CV-)*	
Depth (three-level factor)	*Water depth sampled at the time of the point-count*: *shallow (<25 cm)*, *medium (25–75 cm)*, *deep (>75 cm)*
Mean Depth (covariate)*Mean water depth of the locality*: *values ranging from 1 (shallow) to 3 (deep)*	
Vegetation cover (five different two-level factors)	*Occurrence (presence/absence) of reeds*, *dry/green helophytes and emergent/submerged vegetation*
Mudflats (two-level factor)	*Occurrence (presence/absence) of mudflats at the shore*

### Modelling waterbird occurrence

Species-specific modelling of waterbird occurrence and associations with habitat features and environmental characteristics of wetlands were conducted using two types of statistical models: Generalized Additive Models (GAMs) and Boosted Regression Trees (BRTs). We selected these two methods because GAMs are better at modelling gradual non-linear responses to environmental predictors while BRTs are better at modelling non-gradual responses. Forecasts for each species may vary among modelling techniques, thus by using two very different statistical models (GAM and BRT) we expected to capitalize on their relative strengths [42].

We considered that species occurrence at a point-count is the result of two processes: waterbird presence and detectability (i.e., the species has to be present at the point but must also be detected). We were mainly interested in habitat features that determine waterbird presence but correcting for environmental factors that may influence their detectability at a specific time and location (Table 1). Environmental factors influencing waterbird detectability were studied first by adjusting a number of GAMs with “species richness” (total number of species observed in each point-count) as the response variable. The rationale behind this criterion is that factors influencing waterbird detectability must also have an effect on species richness. As the meteorological variables that could affect waterbird detectability (i.e., sunny weather, presence of clouds, rain and fog, see Table 1) were highly correlated, a Principal Component Analysis (PCA) was performed to reduce the number of dimensions to two axes (PC1 and PC2 accounting for 91% of explained variance). PC1 (82%) mainly distinguished between cloudy (positive values) and sunny days (negative values), whereas PC2 (9%) differentiated between rainy (negative values) and partially cloudy days (positive values). These models indicated that the most relevant predictors of species richness and, therefore, waterbird detectability included observer identity, time of the day (accounting for daily patterns in waterbird activity) and the meteorological PC2 (henceforth called ‘detectability factors’). These parameters were forced in the subsequent species-specific models so that detectability effects were accounted for in the null model before establishing the species association with habitat features.

Some of the habitat features measured in-situ were highly correlated. To avoid issues related to collinearity in the models, a subset of uncorrelated variables was therefore selected based on their ecological relevance and susceptibility to climate-driven changes in water budgets (i.e., increasing evapotranspiration and decreasing precipitation regime). Among environmental predictors, we selected mean water depth, annual mean water salinity, hydroperiod duration, occurrence of muddy areas, aquatic vegetation (emergent, floating and submerged), the percentage of wetlands within a 10-km buffer (hereafter waterbody isolation) and the relative surface flooded (see Table 1 for full description of predictor variables). Models also included the date (days from January 1^st^) to account for seasonal patterns in species abundances, as well as the distance to the coastline to model those waterbirds associated with the coast or with tidal wetlands.

We built GAMs for each single species using the occurrence at a point-count as the response variable. Model fitting started from a null model that included, as explanatory variables, all detectability factors and added new variables following a forward-backward stepwise variable selection procedure based on Akaike Information Criterion (AIC) AIC is known to render large models, but we were interested in building final models that were as simple as possible to examine the environmental variables most clearly related to each species’ occurrence and to avoid the risk of overfitting. Thus, we performed a deviance analysis on the resulting models to retain only those habitat predictors with significant effects once detectability factors were corrected for. BRTs were fitted by including all identified detectability factors and habitat predictors that were found to be significant in GAM models (note that this technique is immune to overfitting). The same GAM procedure was applied to an independent, coarser, but longer time series based on 3614 monthly censuses at 109 different wetlands within the same study area and during four consecutive annual cycles (2004–2009, provided by the “Consejería de Medio Ambiente”; Andalusian government). In this way, we aimed to validate the general results and the robustness of model outputs on habitat associations obtained from our own point-counts (see S1 File). GAMs were fitted in S-Plus 2000 (MathSoft, Inc, USA), whereas BRTs were done in R 2.6.1 [43] with additional functions provided by the R packages gbm [44].

Although two models (a GAM and a BRT) were fitted to each individual species, the full overview of habitat associations for the entire waterbird assemblage was approached by grouping the waterbird species in 7 different guilds (*sensu*, [45]: dabbling ducks, diving birds, fishing birds, large wading birds, raptors, small wading birds and vegetation gleaners; see Table 2 for the full list of species included in each guild).

**Table 2:** List of species considered within guilds. (T) denotes that the species is threatened according to BirdLife International categorization SPEC 1 (European species of global conservation concern), SPEC 2 (species with global population concentrated in Europe and with an unfavourable conservation status in Europe) and SPEC 3 (species not concentrated in Europe, but with an unfavourable conservation status in Europe).

Guild	Spp	Abbreviation	Num
Dabbling ducks	*Anas acuta*	Anaacu (T)	1
	*Anas clypeata*	Anacly (T)	2
	*Anas crecca*	Anacre	3
	*Anas penelope*	Anapen	4
	*Anas platyrhynchos*	Anapla	5
	*Anas strepera*	Anastr (T)	6
	*Anser anser*	Ansans	7
	*Tadorna tadorna*	Tadtad	8
Diving birds	*Aythya ferina*	Aytfer (T)	9
	*Netta rufina*	Netruf	10
	*Oxyura leucocephala*	Oxyleu (T)	11
	*Phalacrocorax carbo*	Phacar	12
	*Podiceps cristatus*	Podcri	13
	*Podiceps nigricollis*	Podnig	14
	*Tachybaptus ruficollis*	Tacruf	15
Fishing birds	*Chlidonias hybrida*	Chlhyb (T)	16
	*Chlidonias niger*	Chlnig (T)	17
	*Larus audouinii*	Laraud (T)	18
	*Larus fuscus*	Larfus	19
	*Larus genei*	Largen (T)	20
	*Larus michahellis*	Larmic	21
	*Larus ridibundus*	Larrid	22
	*Pandion haliaetus*	Panhal (T)	23
	*Sterna albifrons*	Stealb (T)	24
	*Sterna caspia*	Stecas (T)	25
	*Sterna nilotica*	Stenil (T)	26
	*Sterna sandvicensis*	Stesan (T)	27
Large wading birds	*Ardea cinerea*	Ardcin	28
	*Ardea purpurea*	Ardpur (T)	29
	*Ardeola ralloides*	Ardral (T)	30
	*Bubulcus ibis*	Bubibi	31
	*Ciconia ciconia*	Ciccic (T)	32
	*Egretta alba*	Egralb	33
	*Egretta garzetta*	Egrgar	34
	*Ixobrychus minutus*	Ixomin (T)	35
	*Nycticorax nycticorax*	Nycnyc (T)	36
	*Phoenicopterus roseus*	Phoros (T)	37
	*Platalea leucorodia*	Plaleu (T)	38
	*Plegadis falcinellus*	Plefal (T)	39
Raptors	*Circus aeruginosus*	Ciraer	40
	*Milvus migrans*	Milmig (T)	41
	*Milvus milvus*	Milmil (T)	42
Small wading birds	*Actitis hypoleucos*	Acthyp (T)	43
	*Arenaria interpres*	Areint	44
	*Calidris alba*	Calalb	45
	*Calidris alpina*	Calalp (T)	46
	*Calidris ferruginea*	Calfer	47
	*Calidris minuta*	Calmin	48
	*Charadrius alexandrinus*	Chaale (T)	49
	*Charadrius dubius*	Chadub	50
	*Charadrius hiaticula*	Chahia	51
	*Gallinago gallinago*	Galgal (T)	52
	*Glareola pratincola*	Glapra (T)	53
	*Haematopus ostralegus*	Haeost	54
	*Himantopus himantopus*	Himhim	55
	*Limosa lapponica*	Limlap	56
	*Limosa limosa*	Limlim (T)	57
	*Numenius arquata*	Numarq (T)	58
	*Numenius phaeopus*	Numpha	59
	*Pluvialis squatarola*	Plusqu	60
	*Recurvirostra avosetta*	Recavo	61
	*Tringa nebularia*	Trineb	62
	*Tringa ochropus*	Trioch	63
	*Tringa totanus*	Tritot (T)	64
	*Vanellus vanellus*	Vanvan (T)	65
Vegetation gleaners	*Fulica atra*	Fulatr	66
	*Fulica cristata*	Fulcri (T)	67
	*Gallinula chloropus*	Galchl	68
	*Porphyrio porphyrio*	Porpor (T)	69

### Horizon scanning

**Fig 3:**
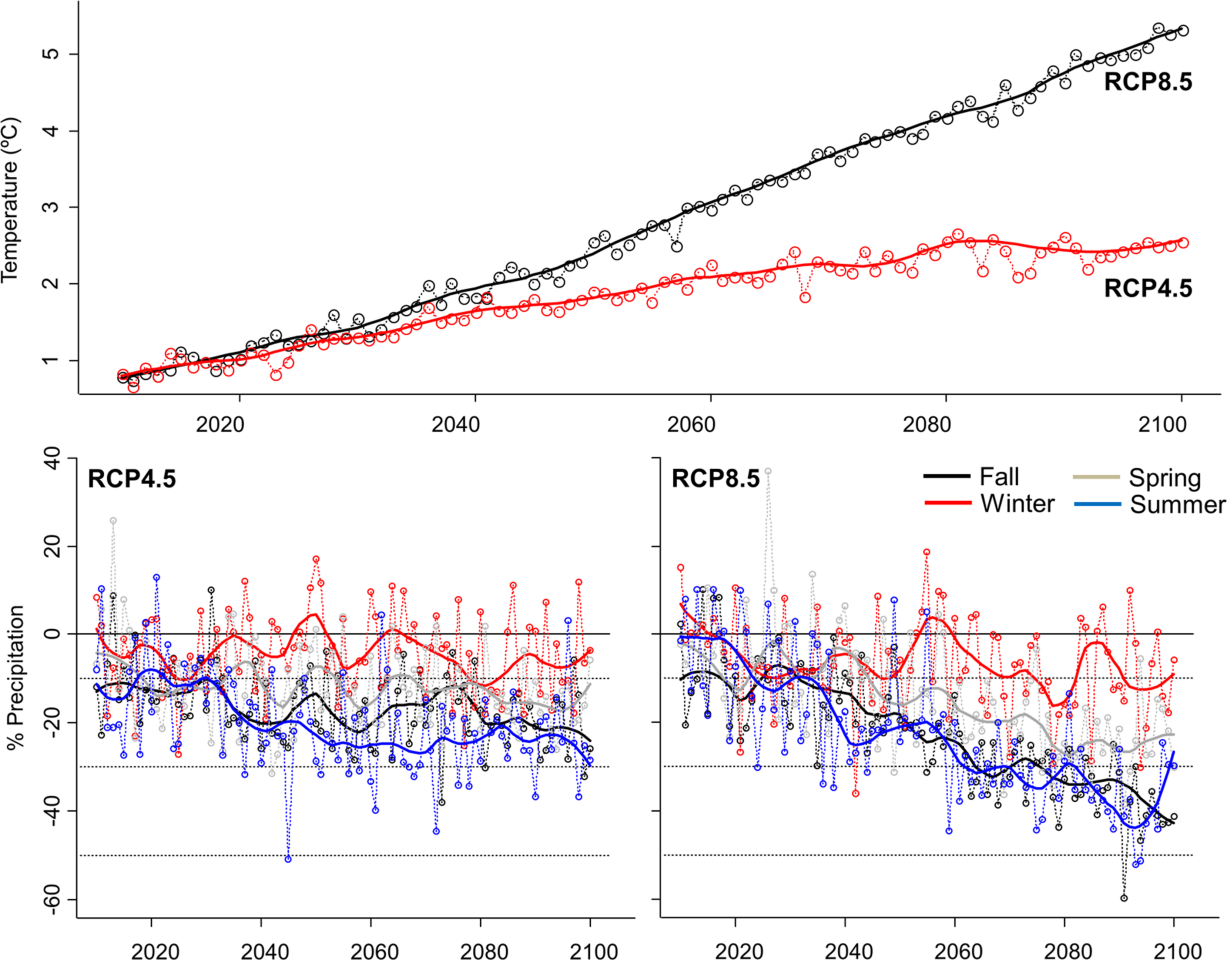
Climate projections. Averaged and smoothed regional projections of climatic variables in the study area (including all available regional models for the provinces of Seville, Cadiz and Huelva; sourced online from AEMET–Agencia Estatal de Meteorología–: http://www.aemet.es/es/serviciosclimaticos/cambio_climat; accessed on March 2017). Trends (2010–2100) for temperature and precipitation are shown for two different Representative Concentration Pathways–RCP–: RCP 8.5 (8.5 W·m^-2^) and RCP 4.5 (4.5 W·m^-2^). Changes in precipitation regimes are split by season. Horizontal dotted lines represent the % change (10%, 30% and 50%) we used for generating the different scenarios in our horizon scanning assessments.

Expected climate-driven changes for the Mediterranean basin will likely affect water budgets in wetlands through increasing temperatures (and hence evapotranspiration) and decreasing precipitation ([17]; see also Fig 3). Overall, this will likely influence wetlands by increasing wetland salinity and waterbody isolation, and decreasing water depth and hydroperiod duration [46,47] and, ultimately, changing the habitat available for waterbirds species. Averaged and smoothed regional projections of climate change in the study area predict a reduction in precipitation and an increase in temperature ranging from 10–50% (Fig 3). Despite the lack of quantitative models linking climate change with environmental predictors for waterbirds, we considered changes in environmental variables of a similar magnitude to those predicted for climatic variables. Accordingly, we generated three different scenarios of 10%, 30%, and 50% of change in salinity, water depth, hydroperiod, and wetland isolation (hereafter, CC10, CC30 and CC50, respectively). We applied these changes to all waterbodies except to tidal and managed wetlands (e.g. saltpans) where water budgets and associate parameters are largely controlled by tidal processes and human activities, respectively.

We used the final GAMs and BRTs for each species to make new predictions of probability of occurrence at the point-count in the three new scenarios. We assumed that the mean probability of occurrence for each waterbird species was a proxy for habitat suitability for that particular species. Climate-driven changes in habitat suitability were then calculated as *(Ps-Po)/Max (Ps*, *Po)*, where Po is the mean probability of occurrence estimated using original habitat predictors, and Ps refers to the mean probability in the new scenario.

## Results

**Fig 4:**
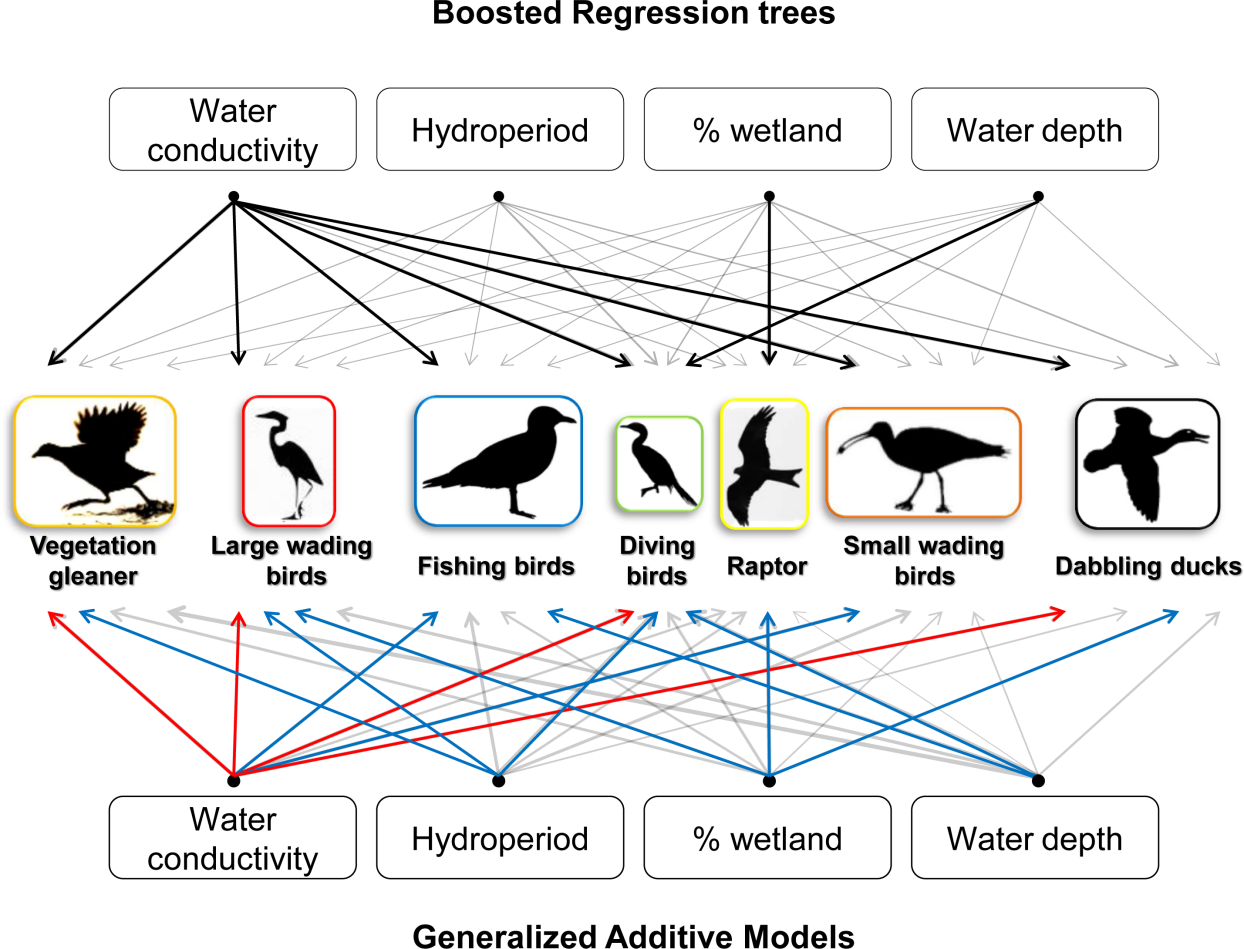
Waterbirds’ associations with environmental features. Waterbird species (n = 69) are grouped into 7 different guilds. Lines connect waterbird guilds with those habitat variables driving their distribution. Those environmental features making up > 15% relative importance for BRT and included in the final GAMs for >80% of species within guilds are highlighted with bold lines. In the case of GAMs, red lines indicate negative effects on respective guilds, whereas blue lines indicate positive effects.

The environmental features that best explain waterbird presence at the point-count were water salinity, water depth, waterbody isolation and hydroperiod duration (Table 3). In turn, these variables were also those most likely to be affected by climate-driven changes in water budgets. However, the GAM estimated coefficients and the BRT relative importance for these key environmental predictors clearly differed among waterbird species and guilds (see S1 and S2 Tables). Overall, water salinity was highlighted as the main environmental predictor for the whole waterbird assemblage regardless of the statistical procedure considered (i.e., GAMs or BRTs; see Table 3, Fig 4 and S1 and S2 Tables). Fishing birds and small wading birds were clearly associated with the highest salinities (tidal areas and salt pans). In contrast, vegetation gleaners and diving birds preferentially used permanent (longer hydroperiods) and deep waterbodies with the lowest salinities (fresh water). Large wading birds inhabit preferentially shallow and permanent waterbodies with a large range of salinities, whereas dabbling ducks mainly occurred in deep but ephemeral waterbodies with intermediate salinities. Finally, raptor distributions were mainly influenced by waterbody isolation. These results were consistent with those obtained from the independent dataset (see S1 File).

**Table 3:** Relative importance of each variable as predictors of waterbird occurrence. For GAMs, we show the percentage of waterbird species (n = 69) for which the predictor was included in the final models. For BRTs, we show the mean relative importance.

	GAMs	BRTs
	% Spp	Mean importance
**Environmental predictors**		
Water salinity	88.41	24.79
Water depth	71.01	8.38
Waterbody isolation	69.57	8.95
Hydroperiod	63.77	2.55
Green helophytes	62.32	1.02
Submerged vegetation	62.32	1.09
Mudflats	62.32	1.63
Relative flooded area	59.42	7.55
Dry helophytes	44.93	0.30
Emergent aquatic vegetation	39.13	0.91
**Geographic predictors**		
Distance to coastline	75.36	12.74

**Fig 5:**
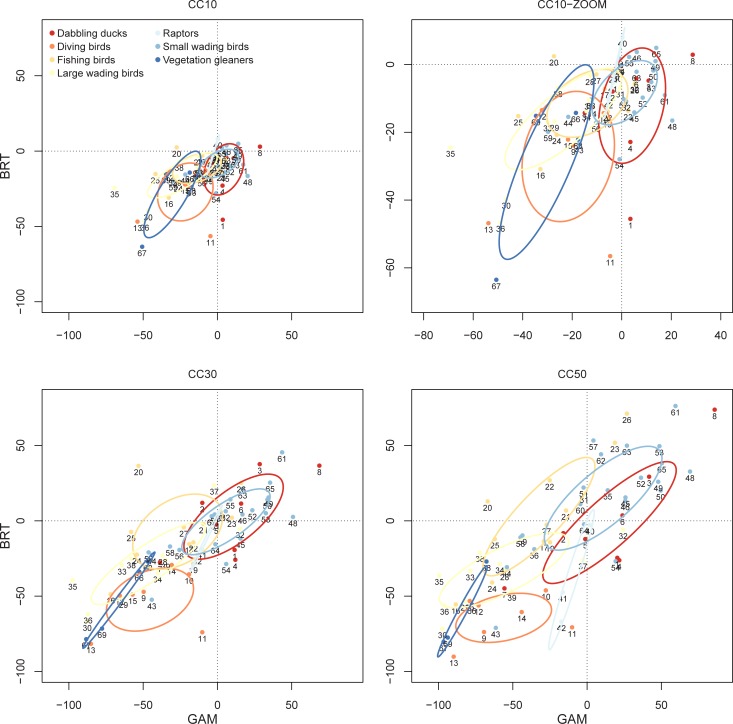
Change in waterbird habitat suitability per guild. We show the effect predicted for three different scenarios with changes of 10%, 30%, and 50% in the main environmental predictors (see Methods). Colours denote the guild and ellipses summarize the distribution of species per guild by considering the variance/covariance matrix. We show the Standard Ellipses corrected for small sample sizes (SEAc) using the R-package SIAR (Parnell et al. 2008). Numeration as in Table 2.

**Fig 6:**
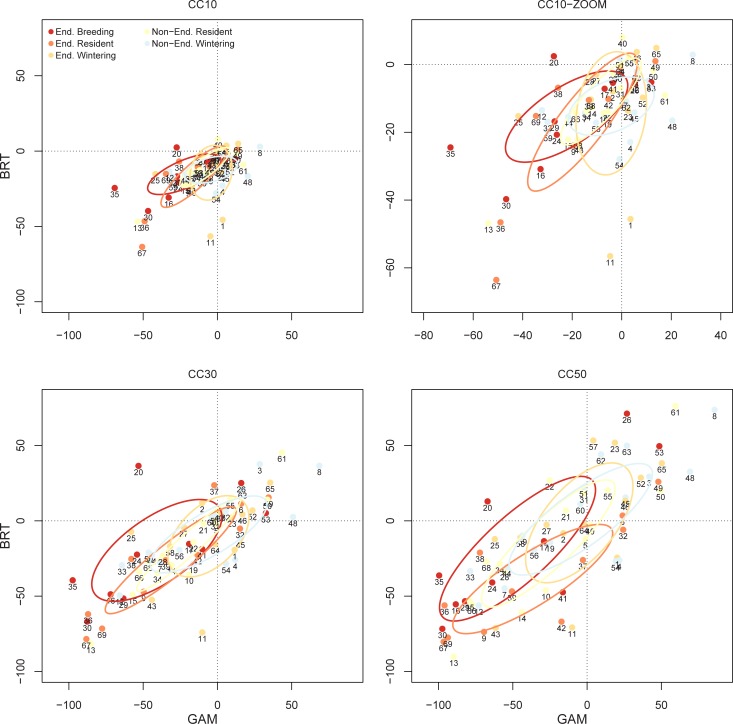
Change in waterbird habitat suitability per life-history strategy and conservation status. We show the effect predicted for three different scenarios with changes of 10%, 30%, and 50% in the main environmental predictors (see Methods). Colours denote waterbird life-history strategy (resident, breeding and wintering) and conservation status (solid lines and solid dots indicate non-endangered species). Ellipses summarize the distribution of species per life-history strategy and conservation status by considering the variance/covariance matrix. We show the Standard Ellipses corrected for small sample sizes (SEAc) using the R-package SIAR (Parnell et al. 2008). Numeration as in Table 2.

Model predictions for habitat suitability agreed between modelling techniques, particularly in the ‘worst’ climatic scenario (Pearson’s r = 0.58, 0.78 and 0.79, for CC10, CC30 and CC50, respectively). Overall, habitat suitability was expected to be negatively impacted by climatic-driven environmental changes for an average of 62% of waterbird species (negative impacts predicted by both GAMs and BRTs), whereas ca. 23% of the species could benefit from future conditions (positive impacts predicted by both GAMs and BRTs, S3 Table and Figs 5 and 6). Diving birds and vegetation gleaners showed the largest reductions in habitat suitability, while small wading birds and dabbling ducks made up the larger proportion of species that could benefit from climate-driven environmental change (Fig 5).

**Fig 7:**
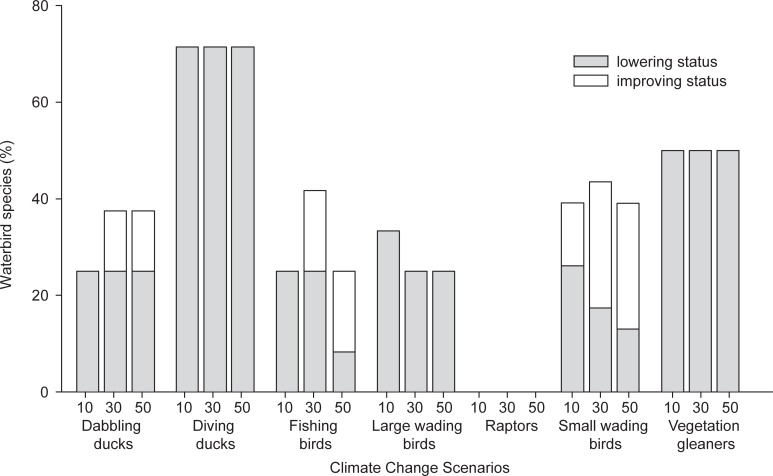
A horizon scan exercise to anticipate conservation issues. Percentage of species per guild whose conservation status may change; i.e. non-endangered species that will be negatively impacted by predicted environmental changes and endangered species that may benefit from the new Climate Change scenarios (CC).

Predicted changes in habitat suitability were on average similar for threatened vs. non-threatened species. In both cases, only 20% of the species may benefit from climate-driven changes in environmental conditions. However, resident species and those that breed in the wetland network (particularly those that are already threatened) will likely be more impacted than wintering species (one-way ANOVA, F_1,89_ = 25.4, p = 0.003, Fig 6). From a horizon-scanning perspective, 71% of currently non-endangered diving birds, 50% of vegetation gleaners and 33% of large wading birds will be negatively impacted within the predicted scenarios. In contrast, ca. 26% of currently threatened, small wading bird species will likely benefit from predicted environmental changes (Fig 7).

## Discussion

Prospective exercises aimed at horizon-scanning human and climate impacts on waterbirds require a thorough comprehension of the environmental drivers structuring their habitats [23,33]. By investigating waterbird distribution in a wetland network in southwestern Spain, we identified a comprehensive set of environmental predictors of habitat use. In particular, several water budget-related environmental traits such as salinity, water depth, water body isolation and hydroperiod, structured the community and were revealed as the most important habitat features predicting species presence. However, various species and guilds showed specific and contrasting responses to different environmental predictors. Accordingly, we should expect that the impact of human and climatic-driven changes in water budgets on waterbirds will be species and guild-specific.

Species and guild-specific traits exert marked morphological and ecological constraints to habitat use, thus resulting in the uneven distribution of waterbirds throughout habitat gradients [33,48–50]. Small wading and fishing birds distributed themselves along the upper range of the water salinity gradient, thus indicating their preferences for coastal and tidal wetlands or saltpans, and demonstrating a tolerance for saline conditions [51]. In contrast, most other species preferentially use low salinity waters. Salty water may be a handicap for these other waterbirds due to dehydration [52] or reduction of feather waterproofing [53]. Water salinity can also create habitat gradients in wetlands by controlling the abundance and composition of primary producers, and hence food availability for herbivorous species like vegetation gleaners and dabbling ducks [33,48,54–56]. Moreover, rainfall and climatic factors largely dominate the hydrological regime in this area, particularly within the Doñana wetland complex [38], with most waterbodies flooding annually due to winter rains and drying up in the summer [30,31,57]. As the drying period progresses, waterbodies will become saltier, but also shallower, and hence less suitable for diving birds [21].

Water depth is also crucial to the feeding success of large wading birds, vegetation gleaners, and dabbling ducks by providing suitable habitats for effective foraging and by controlling the abundance and composition of primary producers [21,58]. Hydroperiod duration is another key factor controlling wetland productivity, with temporary water bodies typically exhibiting poorer fish communities [47], and thus less attractive to large wading and fishing birds. Finally, water body isolation was revealed as another important driver of waterbird presence (see also [49,59]), particularly for highly mobile species, such as raptors that range over large areas [60].

Owing to the contrasting responses of individual waterbird species to habitat predictors, we should expect species-specific changes in habitat suitability as environmental conditions shift towards increasing wetland salinity and isolation, and decreasing depths and hydroperiods. In particular, diving birds, vegetation gleaners, and dabbling ducks will likely face the largest reduction in habitat suitability, whilst many species in the small wading bird guild will likely benefit from the new scenarios (Fig 5). The benefits of changing conditions are already apparent for the Continental European population of black-tailed godwit *Limosa limosa limosa* with an increasing fraction of their otherwise declining overall population [61] wintering in southern Spain instead of in traditional wintering areas in West Africa [27]. In contrast, the steady decline of hydroperiods in our study area [2,30,31] has been related to steep declines in several dabbling ducks preferentially selecting deep waters (e.g., common teal *Anas crecca*, Eurasian wigeon *Anas penelope*; [26], see also [8] for similar trends in wetlands of Eastern Spain), but also with the disappearance of the diving ferruginous duck (*Aythya nyroca*), which was fairly common in the Doñana wetland complex some decades ago [62]. There has also been a decline in the common pochard (*Aythya ferina*), and in the red-crested pochard (*Netta rufina*), which were formerly the two most common diving ducks breeding in Doñana, and are currently uncommon breeders [63]. The crested coot (*Fulica cristata*) is another example of a vegetation gleaner showing a population decline, as has occurred with the white headed duck (*Oxyura leucocephala*), a member of the diving duck guild that became extinct as a breeder in Doñana [64]. The few exceptions to this general agreement with our predictions mainly concern large wading birds, for which habitat suitability is expected to decrease, but whose current population trends are showing a steep increase in breeding numbers [26,36]. In part, this inconsistency may be explained by the ability of these species to exploit alternative, typically man-made habitats (e.g., rice fields; [36,65]) or human related trophic resources that provide individuals with highly efficient feeding opportunities (e.g., refuse from dumps or the introduced Red-swamp Crayfish *Procambarus clarki*; [66–69]).

Climatic impacts may also vary among waterbirds depending on their life-history strategies. In particular, local species (i.e., resident species and those breeding at our study site) will likely be more impacted than wintering waterbirds. These contrasting responses may be even exacerbated by seasonal differences in expected environmental changes, as climate projections predict relatively constant patterns for winter rainfall, but a drastic reduction in the fall, spring and summer precipitation (Fig 3, see also [70]). Moreover, contrasting climate impacts on habitat suitability for wintering and local species will likely be exacerbated in the ‘worst’ of the climate scenarios (i.e., those indicating the highest impacts on water budgets), as most wintering species (ca. 60%) are small wading birds that use muddy areas and open water to forage and will likely benefit from increasing water salinities and reductions in aquatic vegetation.

The European Union–EU–Birds Directive (79/409/ EEC) highlights the need for research and conservation of currently threatened species (see Article 10 and Annex V of the Birds Directive). However, predictive modelling and horizon-scanning exercises are also recognized as a priority to anticipate reliable management and policy decisions for waterbird conservation [71]. From a horizon-scanning perspective, it is worth noting that the conservation status of some species may change, and hence conservation requirements, according to predicted impacts of climate change on habitat suitability. In particular, we identified emerging issues that could have substantial impacts on the conservation of diving birds, vegetation gleaners, or large wading birds (but see above for the inconsistency between our predictions and current population trends for species in the large wading bird guild). Currently, 50% of these species are considered of less concern according to BirdLife International (www.birdlife.org), but changes in habitat suitability are expected to impact them at higher rates than the global average. We therefore encourage early, policy-relevant and practical research on these guilds (see [72].

From the local to the global scale, management actions should consider prospective exercises aimed at disentangling population responses to changing environmental conditions. Here, we modelled waterbird presence as a proxy for habitat suitability through a single annual cycle. However, this parameter does not necessarily reflect all the requirements of different species and associations between environmental variables and waterbird presence could vary interannually. Future studies could extend our assessment of habitat use by focusing on habitat quality, for example by including measures of breeding success, survival rates, body size, and energy intake [33,48], and by confirming that waterbird distribution throughout environmental gradients stands over periods of contrasting environmental conditions. Uncertainty will always be a factor in research on waterbirds and their complex and dynamic systems. The challenge is to use the available data to produce scientifically sound approaches to identifying key issues of waterbird conservation. Such findings may be subsequently readdressed, reevaluated, and even refuted by incorporating additional information whenever available.

Water quality, which has been recognized as one of the most important threats to the Doñana wetland complex [2], should also be accounted for in assessments on habitat quality for waterbirds, as it has being revealed as a clear determinant of their population trends [8,73,74]. Modelled associations with habitat features can also be combined with spatially-explicit information on relevant environmental predictors to derive spatially-explicit predictions on the extent and distribution of suitable habitats for waterbirds [75]. Such spatially-explicit predictions could be periodically updated by incorporating remote-sensing data on environmental conditions [76,77], serving as a formidable addition to the toolbox of ecologists, stakeholders and managers.

## Supporting information

S1 Table Model outputs (GAMs). Estimates for the main environmental predictors obtained from species-specific (69 spp) Generalized Additive Models.
(PDF)

S2 Table Model outputs (BRTs). Relative importance of the main environmental predictors obtained from species-specific (69 spp) Boosted Regression Trees.
(PDF)

S3 Table Changes in habitat suitability. Estimated change in habitat suitability predicted per each species, scenario (CC10, CC30 and CC50) and modelling technique (GAMs and BRTs).
(PDF)

S1 File Habitat associations from an independent dataset. Analyses on habitat associations for the waterbird community using an independent, coarser, but longer time series on monthly surveys within the same study area and for four consecutive annual cycles (2004–2009). These analyses were used to validate the general results and the robustness of model outputs on habitat associations obtained from our own point-counts.
(PDF)
