# Supplementary material for: How will climate change affect endangered Mediterranean waterbirds?
Source: PLoS One. 2018 Feb 13;13(2):e0192702. doi: 10.1371/journal.pone.0192702 (PMC5811028; doi:10.1371/journal.pone.0192702)
Supplement: S1 Table — Estimates for the main environmental predictors obtained from species-specific (69 spp) Generalized Additive Models. (PDF) [file pone.0192702.s001.pdf]

S1 Table. Estimates for main environmental predictors obtained from species-specific (69 spp) Generalized Additive Models. Models exclusively retained those habitat predictors with significant effect on species occurrence (see Material and methods). Thus, empty spaces indicate that these particular variables had no significant effect on species occurrence.

		**Generalized Additive Models**			
**Guild**	**Spp**	**Salinity**	**Depth**	**Isolation**	**Hydroperiod**
Dabbling ducks	*Anas acuta*	-0.034	0.804	3.098	-0.037
	Anas clypeata	-0.04		0.807	
	Anas crecca	-0.03		-1.321	-0.019
	Anas penelope		1.265	2.789	-0.048
	Anas platyrhynchos	-0.019		-0.897	
	Anas strepera	-0.064	0.774	0.632	-0.042
	Anser anser	-0.092		6.251	
	Tadorna tadorna	-0.045	0.932	4.014	
Diving birds	*Aythya ferina*	-0.032	1.352	5.007	-0.036
	Netta rufina	-0.024	4.231		-0.115
	Oxyura leucocephala	0.04			-0.058
	Phalacrocorax carbo	0.016	0.819	-1.228	0.089
	Podiceps cristatus	-0.014	1.918	4.423	0.08
	Podiceps nigricollis	0.041	2.139		-0.026
	Tachybaptus ruficollis	-0.057	0.341	0.787	0.042
Fishing birds	*Chlidonias hybrida*	-0.054		1.541	0.054
	Chlidonias niger			2.975	
	Larus audouinii	0.033	-1.085	-8.773	0.795
	Larus fuscus	0.026	0.913	-1.738	
	Larus genei	0.048	0.637	6.024	0.086
	Larus michahellis	0.059	0.929		
	Larus ridibundus	0.03	0.854		
	Pandion haliaetus	0.029	1.07	1.481	-0.032
	Sterna albifrons	0.025	0.793		0.061
	Sterna caspia	0.028	1.231		0.13
	Sterna nilotica		-0.752	2.122	
	Sterna sandvicensis	0.05	1.854	-4.191	
Large wading birds	*Ardea cinerea*	0.005		1.168	0.035
	Ardea purpurea	-0.116	-0.637	5.342	0.041
	Ardeola ralloides	-0.146		5.501	0.07
	Bubulcus ibis	-0.032	-0.148		
	Ciconia ciconia		0.99		-0.034
	Egretta alba	0.016	-0.99	5.022	0.054
	Egretta garzetta	0.011		0.726	0.046
	Ixobrychus minutus	-0.142		2.722	0.129
	Nycticorax nycticorax	-0.066		4.033	0.072
	Phoenicopterus roseus	0.015	-0.253	3.074	
	Platalea leucorodia			0.922	0.053
	Plegadis falcinellus	-0.077	-1.284	4.327	0.03
Raptors	*Circus aeruginosus*			2.092	-0.007
	Milvus migrans	-0.032	-0.317	3.38	
	Milvus milvus	-0.018	-1.117	5.331	

Small wading birds	*Actitis hypoleucos*		1.304		
	Arenaria interpres	0.041	1.351	2.373	0.052
	Calidris alba	0.041			
	Calidris alpina	0.037	0.446	-0.989	-0.015
	Calidris ferruginea	0.021			0.021
	Calidris minuta	0.029	-0.696		-0.018
	Charadrius alexandrinus	0.047	0.838		-0.05
	Charadrius dubius	-0.025		-5.145	-0.018
	Charadrius hiaticula	0.037	0.926		-0.013
	Gallinago gallinago	-0.034	-0.543	-3.296	
	Glareola pratincola	-0.08	-1.203		
	Haematopus ostralegus	0.046	1.603	-9.216	
	Himantopus himantopus	0.013	-0.198		
	Limosa lapponica	0.029	1.224		
	Limosa limosa		-0.24	1.349	
	Numenius arquata	0.032	1.518		
	Numenius phaeopus	0.039	1.729	-7.233	0.093
	Pluvialis squatarola	0.042	1.602	-1.406	-0.026
	Recurvirostra avosetta	0.039		0.935	-0.032
	Tringa nebularia	0.026	0.223	-0.894	
	Tringa ochropus	-0.019	-0.354	-2.069	
	Tringa totanus	0.041			0.012
	Vanellus vanellus	-0.07	-0.597	0.849	-0.029
Vegetation gleaners	*Fulica atra*	-0.096		1.818	0.062
	Fulica cristata	-0.057	1.133	4.843	0.041
	Gallinula chloropus	-0.078	0.306		0.028
	Porphyrio porphyrio	-0.141	-0.285	2.185	0.073
