# Supplementary material for: How will climate change affect endangered Mediterranean waterbirds?
Source: PLoS One. 2018 Feb 13;13(2):e0192702. doi: 10.1371/journal.pone.0192702 (PMC5811028; doi:10.1371/journal.pone.0192702)
Supplement: S2 Table — Relative importance of the main environmental predictors obtained from species-specific (69 spp) Boosted Regression Trees. (PDF) [file pone.0192702.s002.pdf]

**Table S2.** Relative importance for main environmental predictors obtained from species-specific (69 spp) Boosted Regression Trees.

| | | Boosted regression trees | | | |
|---|---|---|---|---|---|
| **Guild** | **Spp** | **Salinity** | **Depth** | **Isolation** | **Hydroperiod** |
| Dabbling ducks | *Anas acuta* | 22.611 | 3.943 | 14.176 | 5.091 |
| | *Anas clypeata* | 24.125 | 8.738 | 6.863 | 2.955 |
| | *Anas crecca* | 22.571 | 5.963 | 11.164 | 5.702 |
| | *Anas penelope* | 18.608 | 12.387 | 10.453 | 3.142 |
| | *Anas platyrhynchos* | 23.636 | 7.46 | 6.272 | 0.823 |
| | *Anas strepera* | 33.244 | 7.193 | 12.838 | 2.261 |
| | *Anser anser* | 11.762 | 3.881 | 21.272 | 0.649 |
| | *Tadorna tadorna* | 30.819 | 14.244 | 13.281 | 1.285 |
| Diving birds | *Aythya ferina* | 14.887 | 12.344 | 13.724 | 1.75 |
| | *Netta rufina* | 8.94 | 28.72 | 24.999 | 1.778 |
| | *Oxyura leucocephala* | 35.626 | 6.152 | 3.135 | 5.362 |
| | *Phalacrocorax carbo* | 18.221 | 12.642 | 4.746 | 7.037 |
| | *Podiceps cristatus* | 15.659 | 24.773 | 21.236 | 1.217 |
| | *Podiceps nigricollis* | 17.368 | 14.64 | 12.134 | 0.404 |
| | *Tachybaptus ruficollis* | 41.975 | 11.036 | 6.371 | 1.413 |
| Fishing birds | *Chlidonias hybrida* | 16.337 | 4.561 | 4.971 | 1.405 |
| | *Chlidonias niger* | 9.537 | 5.275 | 10.541 | 0.752 |
| | *Larus audouinii* | 47.361 | 2.691 | 4.454 | 0.073 |
| | *Larus fuscus* | 31.75 | 7.892 | 5.572 | 1.37 |
| | *Larus genei* | 47.36 | 8.733 | 2.533 | 0.077 |
| | *Larus michahellis* | 52.495 | 7.884 | 5.515 | 1.068 |
| | *Larus ridibundus* | 31.521 | 9.573 | 7.819 | 3.634 |
| | *Pandion haliaetus* | 22.473 | 11.995 | 6.135 | 1.336 |
| | *Sterna albifrons* | 13.825 | 5.643 | 5.192 | 0.668 |
| | *Sterna caspia* | 27.511 | 8.422 | 7.992 | 0.196 |
| | *Sterna nilotica* | 3.446 | 7.304 | 6.849 | 4.377 |
| | *Sterna sandvicensis* | 28.915 | 4.605 | 13.361 | 0 |
| Large wading birds | *Ardea cinerea* | 13.092 | 10.927 | 7.985 | 8.99 |
| | *Ardea purpurea* | 11.378 | 7.743 | 9.456 | 2.759 |
| | *Ardeola ralloides* | 24.941 | 3.395 | 9.633 | 3.201 |
| | *Bubulcus ibis* | 23.211 | 8.265 | 12.022 | 1.182 |
| | *Ciconia ciconia* | 25.669 | 8.567 | 7.091 | 3.068 |
| | *Egretta alba* | 5.999 | 11.578 | 11.138 | 5.087 |
| | *Egretta garzetta* | 19.579 | 4.524 | 12.696 | 20.124 |
| | *Ixobrychus minutus* | 19.448 | 2.817 | 1.796 | 1.251 |
| | *Nycticorax nycticorax* | 40.809 | 6.04 | 4.834 | 1.978 |
| | *Phoenicopterus roseus* | 14.868 | 14.099 | 17.723 | 4.479 |
| | *Platalea leucorodia* | 15.374 | 8.94 | 10.458 | 4.479 |
| | *Plegadis falcinellus* | 8.241 | 10.075 | 6.628 | 3.393 |
| Raptors | *Circus aeruginosus* | 7.718 | 3.974 | 9.451 | 2.288 |
| | *Milvus migrans* | 10.274 | 3.622 | 18.979 | 0.72 |
| | *Milvus milvus* | 5.406 | 4.914 | 29.765 | 1.146 |
| Small wading birds | *Actitis hypoleucos* | 17.152 | 17.979 | 3.602 | 0.885 |
| | *Arenaria interpres* | 40.374 | 6.144 | 3.599 | 0.003 |
| | *Calidris alba* | 40.938 | 6.41 | 2.979 | 0.398 |
| | *Calidris alpina* | 43.372 | 6.24 | 5.861 | 0.607 |

| | | | | | |
|---|---|---|---|---|---|
| | *Calidris ferruginea* | 14.991 | 5.148 | 4.714 | 0.871 |
| | *Calidris minuta* | 26.007 | 10.719 | 2.983 | 2.393 |
| | *Charadrius alexandrinus* | 40.834 | 6.81 | 5.323 | 1.071 |
| | *Charadrius dubius* | 10.232 | 4.25 | 25.819 | 0.494 |
| | *Charadrius hiaticula* | 40.903 | 5.235 | 2.775 | 0.624 |
| | *Gallinago gallinago* | 12.865 | 2.326 | 10.07 | 2.215 |
| | *Glareola pratincola* | 10.976 | 11.008 | 9.353 | 1.994 |
| | *Haematopus ostralegus* | 17.213 | 3.265 | 1.77 | 0 |
| | *Himantopus himantopus* | 15.077 | 9.746 | 9.212 | 3.924 |
| | *Limosa lapponica* | 17.849 | 9.879 | 1.936 | 0 |
| | *Limosa limosa* | 17.41 | 9.3 | 13.009 | 3.477 |
| | *Numenius arquata* | 51.243 | 11.957 | 8.435 | 0.224 |
| | *Numenius phaeopus* | 37.902 | 10.006 | 11.704 | 0 |
| | *Pluvialis squatarola* | 57.143 | 9.466 | 4.549 | 0.228 |
| | *Recurvirostra avosetta* | 28.64 | 6.565 | 6.744 | 5.562 |
| | *Tringa nebularia* | 30.76 | 4.686 | 7.425 | 4.619 |
| | *Tringa ochropus* | 13.084 | 3.611 | 5.558 | 3.071 |
| | *Tringa totanus* | 44.234 | 4.71 | 5.687 | 5.049 |
| | *Vanellus vanellus* | 10.484 | 15.314 | 5.828 | 3.814 |
| Vegetation gleaners | *Fulica atra* | 45.872 | 8.467 | 5.38 | 3.268 |
| | *Fulica cristata* | 19.252 | 8.053 | 8.784 | 0.193 |
| | *Gallinula chloropus* | 47.495 | 6.079 | 3.5 | 1.61 |
| | *Porphyrio porphyrio* | 39.859 | 6.356 | 7.514 | 9.567 |

| Guild | Spp | Boosted regression trees<br>Salinity | Depth | Isolation | Hydroperiod |
|---|---|---|---|---|---|
| Dabbling ducks | Anaacu | 22.611 | 3.943 | 14.176 | 5.091 |
| | Anacly | 24.125 | 8.738 | 6.863 | 2.955 |
| | Anacre | 22.571 | 5.963 | 11.164 | 5.702 |
| | Anapen | 18.608 | 12.387 | 10.453 | 3.142 |
| | Anapla | 23.636 | 7.460 | 6.272 | 0.823 |
| | Anastr | 33.244 | 7.193 | 12.838 | 2.261 |
| | Ansans | 11.762 | 3.881 | 21.272 | 0.649 |
| | Tadtad | 30.819 | 14.244 | 13.281 | 1.285 |
| Diving birds | Aytfer | 14.887 | 12.344 | 13.724 | 1.750 |
| | Netruf | 8.940 | 28.720 | 24.999 | 1.778 |
| | Oxyleu | 35.626 | 6.152 | 3.135 | 5.362 |
| | Phacar | 18.221 | 12.642 | 4.746 | 7.037 |
| | Podcri | 15.659 | 24.773 | 21.236 | 1.217 |
| | Podnig | 17.368 | 14.640 | 12.134 | 0.404 |
| | Tacruf | 41.975 | 11.036 | 6.371 | 1.413 |
| Fishing birds | Chlhyb | 16.337 | 4.561 | 4.971 | 1.405 |
| | Chlnig | 9.537 | 5.275 | 10.541 | 0.752 |
| | Laraud | 47.361 | 2.691 | 4.454 | 0.073 |
| | Larfus | 31.750 | 7.892 | 5.572 | 1.370 |
| | Largen | 47.360 | 8.733 | 2.533 | 0.077 |
| | Larmic | 52.495 | 7.884 | 5.515 | 1.068 |
| | Larrid | 31.521 | 9.573 | 7.819 | 3.634 |
| | Panhal | 22.473 | 11.995 | 6.135 | 1.336 |
| | Stealb | 13.825 | 5.643 | 5.192 | 0.668 |
| | Stecas | 27.511 | 8.422 | 7.992 | 0.196 |
| | Stenil | 3.446 | 7.304 | 6.849 | 4.377 |
| | Stesan | 28.915 | 4.605 | 13.361 | 0.000 |
| Large wading birds | Ardcin | 13.092 | 10.927 | 7.985 | 8.990 |
| | Ardpur | 11.378 | 7.743 | 9.456 | 2.759 |
| | Ardral | 24.941 | 3.395 | 9.633 | 3.201 |
| | Bubibi | 23.211 | 8.265 | 12.022 | 1.182 |
| | Ciccic | 25.669 | 8.567 | 7.091 | 3.068 |
| | Egralb | 5.999 | 11.578 | 11.138 | 5.087 |
| | Egrgar | 19.579 | 4.524 | 12.696 | 20.124 |
| | Ixomin | 19.448 | 2.817 | 1.796 | 1.251 |
| | Nycnyc | 40.809 | 6.040 | 4.834 | 1.978 |
| | Phoros | 14.868 | 14.099 | 17.723 | 4.479 |
| | Plaleu | 15.374 | 8.940 | 10.458 | 4.479 |
| | Plefal | 8.241 | 10.075 | 6.628 | 3.393 |
| Raptors | Ciraer | 7.718 | 3.974 | 9.451 | 2.288 |
| | Milmig | 10.274 | 3.622 | 18.979 | 0.720 |
| | Milmil | 5.406 | 4.914 | 29.765 | 1.146 |
| Small wading birds | Acthyp | 17.152 | 17.979 | 3.602 | 0.885 |
| | Areint | 40.374 | 6.144 | 3.599 | 0.003 |
| | Calalb | 40.938 | 6.410 | 2.979 | 0.398 |
| | Calalp | 43.372 | 6.240 | 5.861 | 0.607 |

| | | | | | |
|---|---|---|---|---|---|
| | Calfer | 14.991 | 5.148 | 4.714 | 0.871 |
| | Calmin | 26.007 | 10.719 | 2.983 | 2.393 |
| | Chaale | 40.834 | 6.810 | 5.323 | 1.071 |
| | Chadub | 10.232 | 4.250 | 25.819 | 0.494 |
| | Chahia | 40.903 | 5.235 | 2.775 | 0.624 |
| | Galgal | 12.865 | 2.326 | 10.070 | 2.215 |
| | Glapra | 10.976 | 11.008 | 9.353 | 1.994 |
| | Haeost | 17.213 | 3.265 | 1.770 | 0.000 |
| | Himhim | 15.077 | 9.746 | 9.212 | 3.924 |
| | Limlap | 17.849 | 9.879 | 1.936 | 0.000 |
| | Limlim | 17.410 | 9.300 | 13.009 | 3.477 |
| | Numarq | 51.243 | 11.957 | 8.435 | 0.224 |
| | Numpha | 37.902 | 10.006 | 11.704 | 0.000 |
| | Plusqu | 57.143 | 9.466 | 4.549 | 0.228 |
| | Recavo | 28.640 | 6.565 | 6.744 | 5.562 |
| | Trineb | 30.760 | 4.686 | 7.425 | 4.619 |
| | Trioch | 13.084 | 3.611 | 5.558 | 3.071 |
| | Tritot | 44.234 | 4.710 | 5.687 | 5.049 |
| | Vanvan | 10.484 | 15.314 | 5.828 | 3.814 |
| Vegetation gleaners | Fulatr | 45.872 | 8.467 | 5.380 | 3.268 |
| | Fulcri | 19.252 | 8.053 | 8.784 | 0.193 |
| | Galchl | 47.495 | 6.079 | 3.500 | 1.610 |
| | Porpor | 39.859 | 6.356 | 7.514 | 9.567 |
