# Supplementary material for: How will climate change affect endangered Mediterranean waterbirds?
Source: PLoS One. 2018 Feb 13;13(2):e0192702. doi: 10.1371/journal.pone.0192702 (PMC5811028; doi:10.1371/journal.pone.0192702)
Supplement: S3 Table — Estimated change in habitat suitability predicted per each species, scenario (CC10, CC30 and CC50) and modelling technique (GAMs and BRTs). (PDF) [file pone.0192702.s003.pdf]

Table S3. Species-specific change in habitat suitability as predicted for three different scenarios with changes of 10%, 30%, and 50% in the main environmental predictors (see Methods), and based on species-specific habitat associations modelled through Generalized Additive Models (GAMs) and Boosted Regression Trees (BRTs).

	**GAMs**			**BRTs**		
	**CC10**	**CC30**	**CC50**	**CC10**	**CC30**	**CC50**
Actitis hypoleucos	-17.23	-44.10	-61.50	-23.42	-52.46	-71.09
Anas acuta	3.53	11.59	20.31	-45.59	-19.35	-24.49
Anas clypeata	-3.63	-10.22	-15.99	-7.98	11.92	-8.21
Anas crecca	10.86	28.51	41.81	-4.77	37.62	29.34
Anas penelope	3.46	12.02	21.74	-22.91	-25.90	-26.19
Anas platyrhynchos	-0.25	-0.75	-1.22	-0.49	-2.47	-12.06
Anas strepera	5.91	15.96	23.91	-4.08	11.39	3.52
Anser anser	-14.90	-38.62	-55.58	-14.29	-27.67	-44.87
Ardea cinerea	-12.83	-36.81	-55.48	-3.32	-23.10	-33.20
Ardea purpurea	-27.14	-63.25	-82.19	-16.79	-51.58	-53.39
Ardeola ralloides	-46.67	-87.65	-97.39	-39.76	-66.86	-71.68
Arenaria interpres	-21.63	-44.09	-54.02	-15.48	-22.57	-30.80
Aythya ferina	-19.54	-49.69	-69.39	-23.92	-47.08	-73.77
Bubulcus ibis	-0.56	-1.42	-1.97	-6.93	0.46	18.13
Calidris alba	5.09	15.57	25.87	-14.17	-11.83	15.55
Calidris alpina	6.07	16.83	25.73	3.68	4.31	13.32
Calidris ferruginea	-7.87	-20.81	-30.68	-1.67	-4.49	14.08
Calidris minuta	20.44	50.86	69.31	-16.51	2.57	32.66
Charadrius alexandrinus	13.60	34.44	47.96	0.98	15.51	25.92
Charadrius dubius	12.73	33.63	49.49	-1.75	14.07	20.10
Charadrius hiaticula	-0.77	-1.86	-2.34	1.74	4.29	22.15
Chlidonias hybrida	-32.80	-71.84	-88.39	-30.89	-48.76	-55.43
Chlidonias niger	-6.90	-19.00	-29.04	-7.17	-15.36	-13.53
Ciconia ciconia	5.25	15.25	24.32	-5.56	-5.18	-5.92
Circus aeruginosus	0.32	1.10	2.03	8.04	6.08	-2.80
Egretta alba	-29.94	-64.21	-78.72	-16.96	-29.48	-33.31
Egretta garzetta	-14.24	-40.25	-58.72	-13.78	-34.64	-28.59
Fulica atra	-18.55	-52.65	-77.58	-14.27	-33.69	-55.57
Fulica cristata	-50.68	-88.23	-96.61	-63.55	-78.49	-80.29
Gallinago gallinago	8.49	23.58	36.23	-9.78	6.98	28.65
Gallinula chloropus	-17.64	-46.76	-67.77	-22.16	-27.05	-27.01
Glareola pratincola	11.96	32.60	48.66	-5.23	5.12	49.55
Haematopus ostralegus	-0.89	5.86	19.20	-27.93	-28.58	-26.88
Himantopus himantopus	3.02	8.70	13.90	2.22	14.28	20.35
Ixobrychus minutus	-69.15	-97.45	-99.57	-24.47	-39.48	-36.27
Larus audouinii	-19.80	15.41	39.74	-35.25	-42.88	-28.10
Larus fuscus	-5.83	-16.26	-24.95	-15.80	-28.48	-14.26
Larus genei	-27.40	-53.11	-66.98	2.45	36.49	12.88
Larus michahellis	-3.38	-9.34	-14.22	-2.60	-2.06	6.86
Larus ridibundus	-5.68	-16.11	-25.17	-13.91	-14.18	26.90

Limosa lapponica	-10.35	-25.61	-35.54	-17.17	-19.34	-18.90
Limosa limosa	0.68	2.29	4.22	-10.20	5.65	53.32
Milvus migrans	-3.56	-10.26	-16.27	-5.41	-18.71	-47.43
Milvus milvus	-5.34	-13.40	-17.22	-10.18	-22.62	-66.98
Netta rufina	-7.29	-19.07	-27.73	-14.37	-35.57	-46.12
Numenius arquata	-12.49	-31.83	-44.84	-10.34	-16.98	-11.06
Numenius phaeopus	-29.80	-46.34	-43.48	-19.86	-21.02	-9.72
Nycticorax nycticorax	-48.95	-87.06	-95.96	-46.57	-61.92	-56.11
Oxyura leucocephala	-4.66	-10.19	-10.08	-56.55	-74.03	-70.78
Pandion haliaetus	2.56	9.74	18.68	-13.63	2.18	51.84
Phalacrocorax carbo	-32.27	-65.71	-72.96	-13.50	-49.53	-56.20
Phoenicopterus roseus	-0.85	-2.12	-2.80	-1.64	23.64	-26.03
Platalea leucorodia	-25.74	-57.97	-72.15	-6.87	-25.28	-21.10
Plegadis falcinellus	-13.26	-34.80	-50.59	-10.53	-26.20	-46.85
Pluvialis squatarola	-1.63	-3.88	-4.53	-2.33	3.13	11.10
Podiceps cristatus	-53.82	-85.22	-89.82	-46.83	-81.62	-90.16
Podiceps nigricollis	-11.95	-30.81	-44.03	-12.69	-29.29	-60.55
Porphyrio porphyrio	-34.62	-77.64	-93.70	-15.19	-71.49	-77.50
Recurvirostra avosetta	17.44	43.53	59.54	-9.07	45.41	76.20
Sterna albifrons	-26.24	-54.15	-63.84	-20.73	-22.40	-40.91
Sterna caspia	-41.75	-58.03	-62.05	-15.23	-7.31	-12.15
Sterna nilotica	5.27	16.08	26.85	-5.79	25.11	71.18
Sterna sandvicensis	-10.21	-22.55	-27.23	-2.94	-4.28	-2.65
Tachybaptus ruficollis	-21.70	-57.06	-78.96	-22.16	-49.01	-53.28
Tadorna tadorna	28.67	68.62	85.84	2.89	36.59	73.71
Tringa nebularia	1.84	5.60	9.41	-10.88	6.37	44.10
Tringa ochropus	5.99	17.02	26.79	-2.15	21.03	49.92
Tringa totanus	-0.52	-1.39	-2.05	-0.23	-15.54	-2.29
Vanellus vanellus	13.99	35.53	50.34	4.88	25.38	38.27
