# Supplementary material for: How will climate change affect endangered Mediterranean waterbirds?
Source: PLoS One. 2018 Feb 13;13(2):e0192702. doi: 10.1371/journal.pone.0192702 (PMC5811028; doi:10.1371/journal.pone.0192702)

Habitat associations from an independent dataset

An independent dataset was used for testing the robustness of model outputs obtained from our own point-counts along a single season. This dataset was provided by "Consejería de Medio Ambiente" – CMA – (Andalusian government) and included information on waterbird occurrences from 3614 monthly surveys at 109 different waterbodies (Fig. S1). This dataset might be considered coarser than the one from our own censuses as CMA surveys were not conducted using a standardized methodology (based on estimates for total abundances per species) and survey effort was not controlled. Further, no information on drivers of waterbird detectability (e.g. observer, day time, meteorology, see Table 1) was available. Finally, in situ data on main environmental predictors of waterbird occurrences were scarce, so that we were obligated to resort to general data from official reports in most cases. Despite these considerations, CMA surveys spread over 4 different annual cycles (from January 2004 to December 2007), thus having the potential for providing strong enough conclusions on habitat associations for the entire waterbird assemblage.

A forward selection procedure based on Generalized Additive Models (GAMs) and the Akaike's information criterion (AIC) was used to model waterbird occurrences from CMA surveys. Species-specific GAMs included mean water depth, salinity and the percentage of wetlands within a 10 km buffer as main environmental predictors (Table 1). The date (days from January 1st) and year were also included to account for seasonal and interannual patterns in species abundances.

Considered environmental predictors were retained in most species-specific models (water salinity: 90% of species; water depth: 87%; % wetlands: 86%). Overall, model outputs from CMA surveys (Table 2) were consistent with those from our own

census. In particular, both outputs fully agree when considering the effect (positive or negative) of water salinity and water depth on the occurrence of the 7 different guilds: dabbling ducks, diving birds, fishing birds, large wading birds, raptors, small wading birds and vegetation gleaners. In the case of % wetland, model outputs were consistent for 5 of the 7 guilds. Fig. S2 summarizes the relationships between habitat variables and waterbird guilds when considering the two independent datasets.

Table 1. Complete list of predictors and control factors considered for modelling habitat associations in the waterbirds community from south-west Spain using surveys from “Consejería de Medio Ambiente” – CMA – (Andalusian government)

**WATERBIRD OCCURRENCE**	
**Control factors**	
Date (covariate -4 d.f. spline-)	*Days from January 1st, to account for seasonal changes in the occurrences of migratory waterbirds*
Year (covariate -1 to 3 d.f. spline-)	*Correlative nominal value to account for inter-annual trends in the abundance of species*
**Geographic predictor**	
Geographic locations (two covariates)	*Latitude and longitude*
Distance to coastline (covariate)	*minimum Euclidean distance to the coastline*
**Environmental predictor**	
Isolation (covariate)	*% of wetlands within a 10 km buffer from the census site*
Salinity (covariates)	*water salinity (sourced from official reports)*
Mean Depth (covariate)	*mean water depth (sourced from official reports): values ranging from 1 (shallow) to 3 (deep)*

Table 2. Estimates from species-specific Generalized Additive Models that considered data on waterbird occurrences from CMA surveys. Abbreviations as in Table 2

		Generalized Additive Models		
**Guild**	**Spp**	**Salinity**	**Depth**	**Isolation**
Dabbling ducks	Anaacu	-0.0093	0.1618	0.0228
	Anacly	-0.0063		0.0150
	Anacre	-0.0187	0.2983	0.0109
	Anapen		0.7256	0.0236
	Anapla	-0.0170	0.1631	
	Anastr	-0.0205	0.1030	0.0113
	Ansans	-0.0076		0.0341
	Tadtad	-0.0191	-0.3938	0.0100
Diving birds	Aytfer	-0.0159	-0.6628	0.0148
	Netruf	-0.0208	-1.1753	
	Oxyleu	0.0264	1.1948	0.0453
	Phacar	0.0091	1.1214	
	Podcri	-0.0094	0.9426	0.0216
	Podnig		-0.2372	0.0123
	Tacruf	-0.0308	-0.2598	-0.0060
Fishing birds	Alcatt	0.0084	0.9012	0.0071
	Chlhyb	-0.0057		0.0142
	Chlnig	0.0186	1.1154	0.0368
	Laraud	0.0771	4.1059	0.0233
	Larfus	0.0211	1.1257	
	Largen	0.0587	0.6603	0.0803
	Larmic	0.0234	0.8919	-0.0048
	Larrid	0.0247	0.6456	
	Panhal	0.0167	1.2759	0.0235
	Stealb	0.0274	0.5067	0.0179
	Stecas	0.0385	0.7902	0.0251
	Stenil			
	Stesan	0.0560	1.8225	0.0156
Large wading birds	Ardcin	-0.0041	0.4916	0.0047
	Ardpur	-0.0192	0.3087	0.0115
	Ardral	-0.0324		
	Bubibi		0.2186	-0.0057
	Ciccic		0.1553	0.0054
	Cicnig	-0.0047	-1.0624	0.0195
	Egralb		-0.4582	0.0260
	Egrgar	0.0079	0.4706	
	Ixomin			
	Nycnyc	-0.0196		0.0151
	Phoros	0.0224	-0.3145	0.0215
	Plaleu	0.0069	0.3365	0.0122

Guild	Spp	Generalized Additive Models Salinity	Depth	Isolation
	Plefal	-0.0247	-0.9701	0.0195
Raptors	Ciraer	-0.0125	-0.2951	0.0169
	Milmig	-0.0117	-0.9628	0.0347
	Milmil			
Small wading birds	Acthyp	0.0077	0.4183	0.0035
	Areint	0.0404	1.1762	0.0389
	Calalb	0.0515	2.1161	0.0152
	Calalp	0.0313	0.8139	0.0118
	Calcan	0.0468	1.8683	0.0518
	Calfer	0.0208	0.7383	0.0234
	Calmin	0.0234	0.6070	0.0182
	Chaale	0.0371	0.8272	0.0053
	Chadub	-0.0081	0.5874	
	Chahia	0.0235	0.7862	0.0039
	Galgal		-0.3893	
	Glapra	0.0466	2.0428	0.0725
	Haeost	0.0159	0.1110	0.0035
	Himhim	0.0513	2.0523	0.0527
	Limlap	0.0120	0.3874	0.0210
	Limlim	0.0441	1.5509	0.0436
	Numarq	0.0418	1.4641	0.0444
	Numpha			0.0171
	Phipug	0.0448	1.2583	0.0285
	Plusqu	0.0206	0.1410	0.0192
	Recavo	0.0112	0.5543	0.0275
	Triery	0.0199	0.2428	0.0107
	Trineb	-0.0072	-0.2031	0.0072
	Trioch	0.0267	0.3744	0.0123
	Tritot	-0.0066	-0.5657	0.0149
	Vanvan	-0.0346		0.0057
Vegetation gleaners	Fulatr	-0.0225		0.0079
	Fulcri	-0.0222	0.1452	-0.0054
	Galchl	-0.0072		
	Porpor	-0.0330	-0.1060	0.0041

Figure S1. Distribution of point-count localities (black dots) surveyed during CMA censuses. Point counts were carried out monthly at 109 different waterbodies during the 2004-2007 annual cycles. The wetland network includes the Tinto & Odiel marshes (1), the Doñana wetland complex (2) and Bay of Cadiz (3).

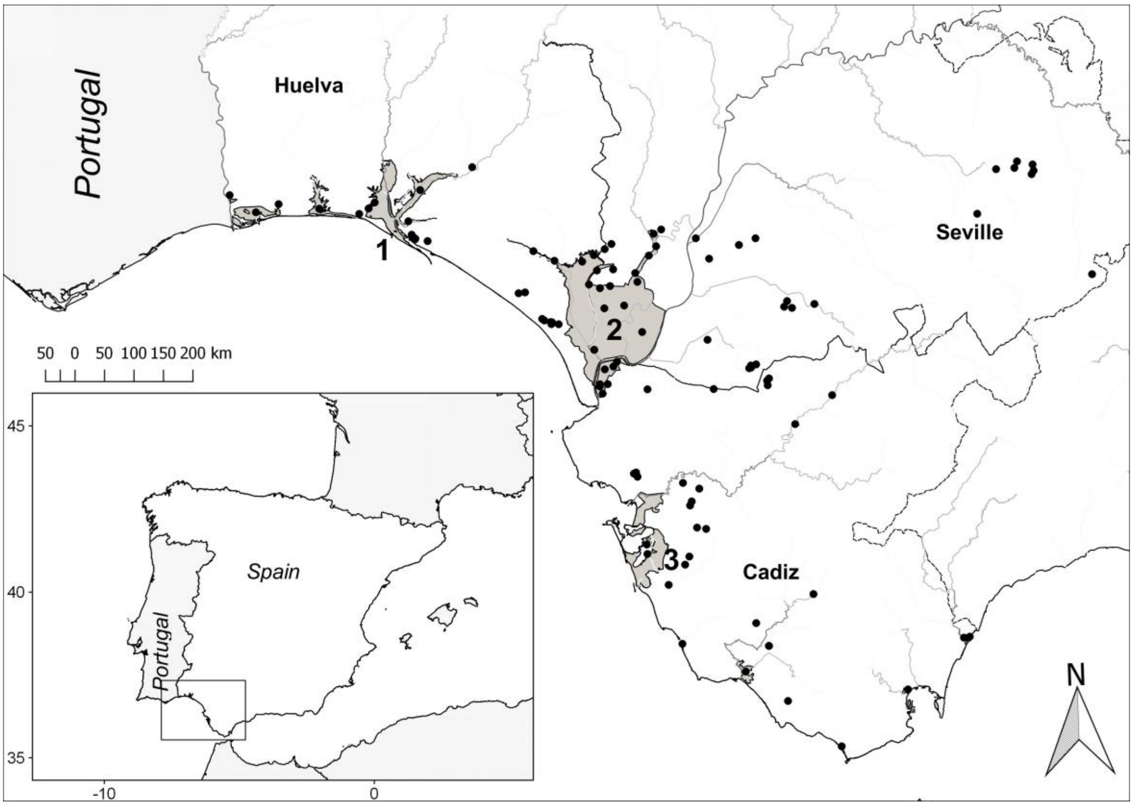

Figure S2. Waterbirds' associations with habitat features obtained from two independent datasets: the finer and short-term (2008) dataset obtained through own censuses, and the coarser, but longer-term (2004-2007) data provided by CMA. Waterbird species are grouped in 7 different guilds. Arrows represent the effect of the most important environmental predictors (those retained in more than 80% of species-specific models). Red lines indicate negative effects to respective guilds, whereas blue lines indicate positive effects.

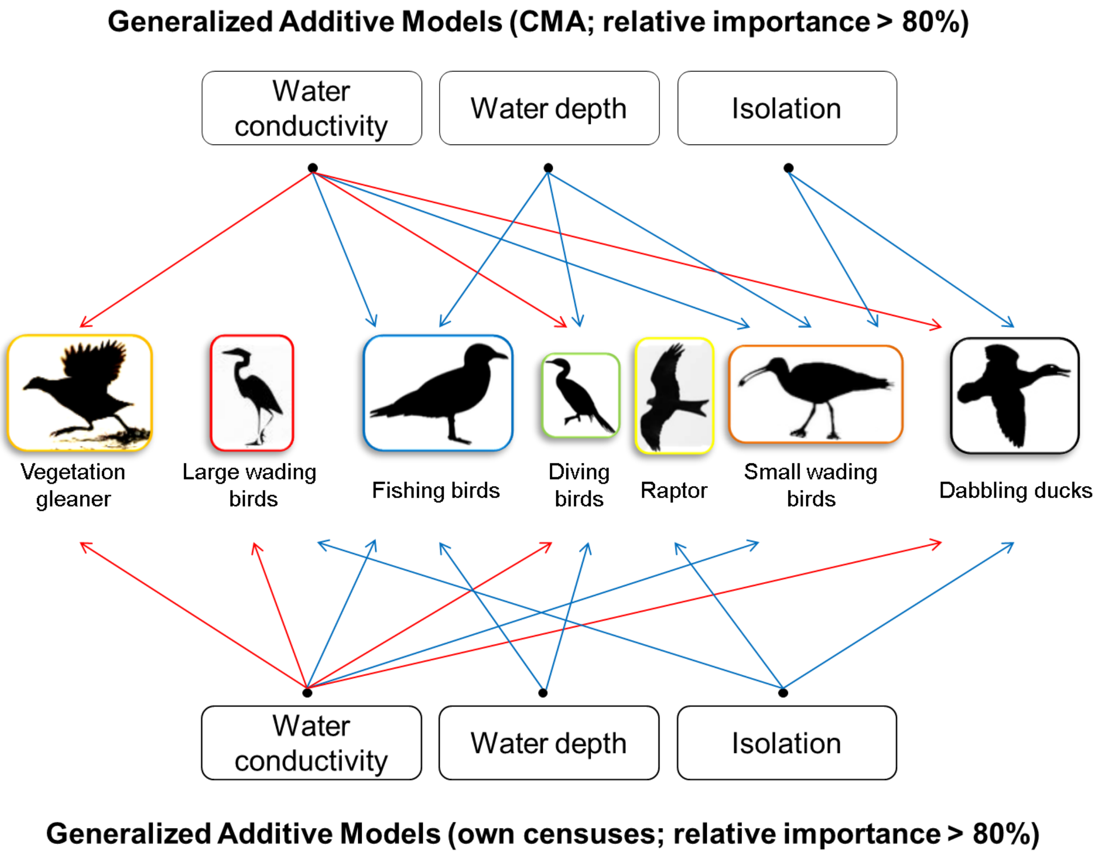

Supplement: S1 File — Analyses on habitat associations for the waterbird community using an independent, coarser, but longer time series on monthly surveys within the same study area and for four consecutive annual cycles (2004–2009). These analyses were used to validate the general results and the robustness of model outputs on habitat associations obtained from our own point-counts. (PDF) [file pone.0192702.s004.pdf]
